# Effective, homogeneous and transient interference with cytosine methylation in plant genomic DNA by zebularine

**DOI:** 10.1111/j.1365-313X.2008.03699.x

**Published:** 2008-10-30

**Authors:** Tuncay Baubec, Ales Pecinka, Wilfried Rozhon, Ortrun Mittelsten Scheid

**Affiliations:** Gregor Mendel Institute of Molecular Plant Biology, Austrian Academy of SciencesDr Bohr-Gasse 3, 1030 Vienna, Austria

**Keywords:** DNA methylation, methylation inhibitor, zebularine, epigenetic regulation, transcriptional reactivation, Arabidopsis

## Abstract

Covalent modification by methylation of cytosine residues represents an important epigenetic hallmark. While sequence analysis after bisulphite conversion allows correlative analyses with single-base resolution, functional analysis by interference with DNA methylation is less precise, due to the complexity of methylation enzymes and their targets. A cytidine analogue, 5-azacytidine, is frequently used as an inhibitor of DNA methyltransferases, but its rapid degradation in aqueous solution is problematic for culture periods of longer than a few hours. Application of zebularine, a more stable cytidine analogue with a similar mode of action that is successfully used as a methylation inhibitor in *Neurospora* and mammalian tumour cell lines, can significantly reduce DNA methylation in plants in a dose-dependent and transient manner independent of sequence context. Demethylation is connected with transcriptional reactivation and partial decondensation of heterochromatin. Zebularine represents a promising new and versatile tool for investigating the role of DNA methylation in plants with regard to transcriptional control, maintenance and formation of (hetero-) chromatin.

## Introduction

**Figure 1:**
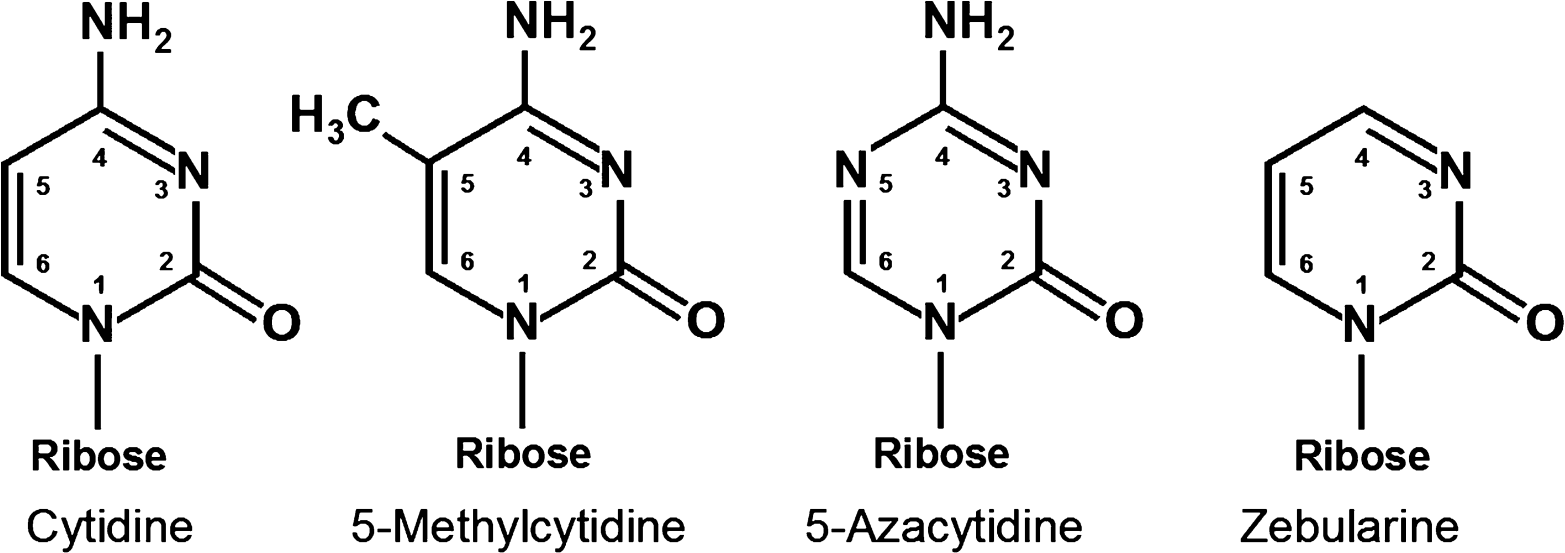
Chemical structure of cytidine, its methylated form, 5-methylcytidine and the methylation inhibitors 5-azacytidine and zebularine (adapted from Cheng *et al.*, 2003).

Post-replicative modification of genomic DNA at the 5C position by methylation of cytosine residues (^m^C) is widespread, though not universal, across a broad range of organisms. In those species that display it, DNA methylation is an important hallmark of epigenetic regulation, coupling additional, potentially heritable information to the genetic information while preserving the original DNA sequence. DNA methylation is enzymatically established by DNA methyltransferases and can cause direct transcriptional repression or an indirect effect via binding of specific proteins. In contrast to evolutionary relationships, DNA methylation and its interpretation in mammals seems to be more similar to that found in higher plants than in any other animal class. In both groups, the level of methylated cytosines is significant, its location is specific, the group of proteins interacting with the modification is diverse and correct DNA methylation is required for regular development. Experimental interference with establishing or maintaining DNA methylation has a considerable and complex impact on vigour, morphology or gene expression, as observed with methyltransferase knockout or knockdown techniques (Finnegan *et al.*, 1996; Li *et al.*, 1992; Okano *et al.*, 1999; Ronemus *et al.*, 1996; Vongs *et al.*, 1993). Manipulation of DNA methylation has also been achieved by modification of target sequences (Dieguez *et al.*, 1997; Klug and Rehli, 2006) or by specific inhibitors (for review see Lyko and Brown, 2005; and Yoo and Jones, 2006). While genetic modification of methylation is usually extensive and permanent, inhibitor treatments allow for partial and transient induction of methylation changes. Chemical analogues of cytosine which are incorporated into DNA are widely used inhibitors. They form covalent adducts with DNA methyltransferases, limiting their further catalytic activity (Santi *et al.*, 1983) and thereby reducing overall DNA methylation. 5-Azacytidine (5-aza) and 5-aza-2′-deoxycytidine (decitabine) are especially commonly applied inhibitors in plants and animals. Both induce hypomethylation, transcriptional reactivation and developmental effects in plant and animal systems, and have gained special attention as cancer therapeutics for malignancies that are based on erratic hypermethylation of tumour suppressor genes (for review see Christman, 2002; and Yoo and Jones, 2006). However, both drugs are extremely unstable in aqueous solution (Beisler, 1978; Constantinides *et al.*, 1977), making administration of defined doses difficult under physiological conditions. Further, both drugs have high toxicity and many side-effects (Ghoshal and Bai, 2007). The search for more stable and less toxic methylation inhibitor drugs has led to the identification of zebularine (1-(β-d-ribofuranosyl)-1,2-dihydropyrimidine-2-one; Figure 1) as a potent drug (Cheng *et al.*, 2003; Marquez *et al.*, 2005; Yoo and Jones, 2006; Yoo *et al.*, 2004; Zhou *et al.*, 2002), originally developed as a cytidine deaminase inhibitor. Acting in a similar way as 5-aza and decitabine, zebularine has a much longer half-life under physiological conditions and fewer side-effects (Cheng *et al.*, 2003). Its action in cancer models has been proven in several studies (Herranz *et al.*, 2006; Marquez *et al.*, 2005; Rao *et al.*, 2007; Scott *et al.*, 2007), although clinical trials have not yet been performed (Yoo and Jones, 2006). Given the limitations of 5-aza instability and toxicity in plant research applications as well (Weber *et al.*, 1990), and the original discovery of the demethylating and reactivating effect of zebularine in the filamentous fungus *Neurospora* (Cheng *et al.*, 2003), it is surprising that as far as we are aware no study has so far addressed the effect of zebularine on plant DNA.

We present data on DNA demethylation in the genomic DNA of *Arabidopsis thaliana* and *Medicago sativa* after application of different doses of zebularine and lengths of treatment. Furthermore, we compare the overall levels of ^m^C as well as ^m^C in different sequence contexts after zebularine treatment at transgenic and endogenous single-copy and repetitive sequences, and analyse the effect on transcriptional activity. The data show that zebularine is a potent dose-dependent and non-discriminative inducer of hypomethylation and transcription, and is a suitable tool for investigating the important role of DNA methylation in plants.

## Results

### Zebularine induces dose-dependent and transient growth inhibition

**Figure 2:**
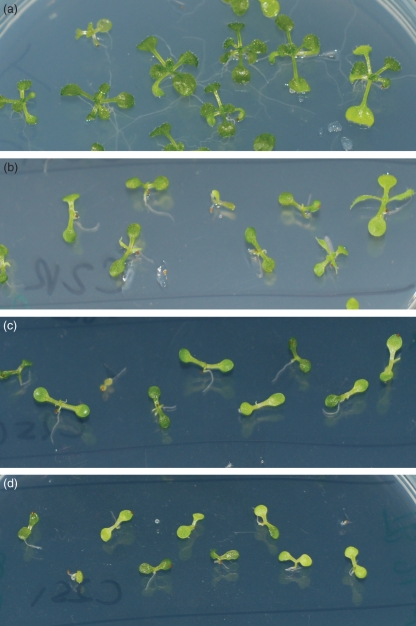
Zebularine treatment affects plant growth and development Arabidopsis seedlings grown for 14 days on zebularine-containing medium with (a) 0 μm, (b) 20 μm, (c) 40 μm or (d) 80 μm zebularine. Images were taken 14 days after sowing.

Since reduced DNA methylation results in abnormal plant development (Finnegan *et al.*, 1996; Jeddeloh *et al.*, 1998; Mathieu *et al.*, 2002; Ronemus *et al.*, 1996), the concentration range of potential effects of zebularine as a methylation inhibitor was established by scoring for its phenotypic effects on plant development. *Arabidopsis thaliana* (accession Zürich) was grown on media containing 0, 20, 40 and 80 μm zebularine (Figure 2a–d). Minor developmental retardation was observed 14 days after germination (dag) at a concentration as low as 20 μm zebularine (Figure 2b). The plants grew secondary roots, but were slightly delayed in growth and developed elongated true leaves when compared with mock-treated plants (Figure 2a). At 40 μm zebularine, true leaves did not expand and roots were much shorter (Figure 2c) than observed at 20 μm. At 80 μm zebularine, plants showed severe inhibition of growth; they did not develop beyond the cotyledon stage and had severely affected root growth (Figure 2d). Nevertheless, the majority of zebularine-treated plants from all concentrations could be rescued by transferring them after 14 or 21 days of treatment to inhibitor-free growth medium. Rescued plants showed complete recovery and a normal seed set. Therefore, transient exposure to zebularine concentrations up to 80 μm causes growth effects that indicate effectiveness and allow subsequent recovery of fertile plants after the treatment.

### Zebularine causes a dose-dependent and transient reduction of global 5-methyldeoxycytidine levels in plants

**Figure 3:**
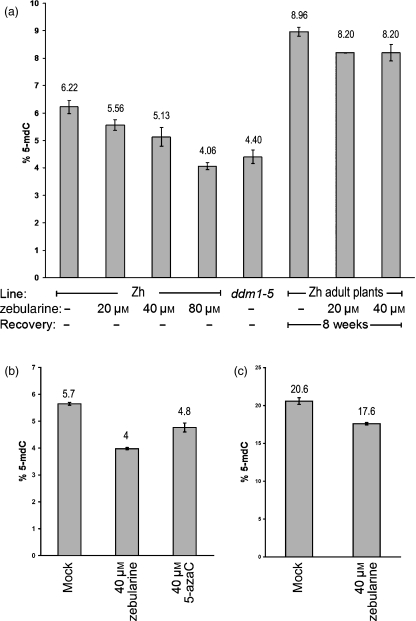
Global levels of 5-methyldeoxycytidine (5-mdC) are reduced by zebularine treatment. (a) Genomic DNA extracted from mock-treated Arabidopsis seedlings or seedlings grown on 20–80 μm zebularine were analysed in triplicate for 5-mdC content by HPLC. The 5-mdC levels were compared with *ddm*1-5 mutant seedlings and adult plants after 8 weeks’ recovery. Zh, wild-type accession Zürich. (b) Zebularine reduces the global level of 5-mdC in *Arabidopsis thaliana* accession Zürich even more than the same concentration of 5-azacytidine (5-azaC; 40 μm, same protocol). (c) Zebularine reduces 5-mdC levels in *Medicago sativa*.

To investigate the effect of the drug treatment on the overall levels of 5-methyldeoxycytidine (5-mdC), mock- and zebularine-treated plants were compared with plants in which DNA methylation was reduced by genetic means. Mutations in the *DDM*1 gene drastically decrease the level of 5-mdC (Jeddeloh *et al.*, 1999; Vongs *et al.*, 1993). Plants were germinated and grown for 21 days on media containing 0, 20, 40 or 80 μm zebularine prior to preparation of genomic DNA. Global 5-mdC levels were analysed as a percentage of 5-mdC in relation to total deoxycytidine (dC) levels using cation exchange HPLC (Rozhon *et al.*, 2008). Mock-treated wild-type seedlings (accession Zürich) had 6.2% 5-mdC, whereas the level was reduced to 4.4% in *ddm*1-5 seedlings, which is in agreement with previously published values (Leutwiler *et al.*, 1984; Rozhon *et al.*, 2008). Levels of 5-mdC in zebularine-treated seedlings were also significantly decreased in a dose-dependent manner, ranging from 5.6, 5.1 to 4.0% in plants treated with 20, 40 and 80 μm zebularine, respectively (Figure 3a). Therefore, zebularine can induce significant hypomethylation similar to genetically achieved levels.

We also analysed global 5-mdC levels in DNA from the leaf tissue of adult plants grown for 8 weeks without an inhibitor, following the initial 21-day treatment with 0, 20 and 40 μm zebularine. DNA from all mature leaf samples had 1.4–1.6-fold more 5-mdC than seedlings, reflecting the developmental changes of DNA methylation levels previously described for untreated plants (Rozhon *et al.*, 2008; Ruiz-Garcia *et al.*, 2005). The difference between mock- and zebularine-treated adult plants decreased to insignificant values (Figure 3a), in agreement with the phenotypic recovery. Therefore, zebularine-induced reduction in 5-mdC levels, even at levels similar to genetically caused hypomethylation, is transient and can be overcome, at least globally, by growth in the absence of the drug.

To compare the efficiency of zebularine with the commonly applied but less stable DNA methylation inhibitor 5-aza, wild-type plants were germinated and grown for 21 days side-by-side on freshly prepared 0 or 40 μm zebularine- or 5-aza-containing media and analysed for the global 5-mdC levels as described. These were decreased in zebularine-treated plants to 4.0% (±0.04) and upon 5-aza treatment to 4.8% (±0.17) (Figure 3b). Therefore, zebularine is as efficient as, if not more so, than the commonly applied inhibitor 5-aza.

To test whether zebularine is effective in plant species other than *A. thaliana*, 5-mdC levels of *M. sativa* seedlings either mock-treated or treated with 40 μm zebularine for 1 week were analysed using the method described above. Mock-treated *Medicago* had 20.6% (±0.44) 5-mdC as previously reported (Rozhon *et al.*, 2008), whereas zebularine-treated *Medicago* had only 17.6% (±0.16) 5-mdC (Figure 3c). This indicates that zebularine is also a potential inhibitor of DNA methylation in other plant species.

### Zebularine causes transient hypomethylation at transcriptionally inactive repeats

In order to elucidate whether the zebularine-induced DNA hypomethylation would affect different genomic regions in the same or in distinct ways, we conducted Southern blot experiments using methylation-sensitive restriction enzymes and sequence-specific probes homologous to different endogenous target sites known to be methylated. These included repetitive sequences such as *Athila*-related transcriptionally silent information (TSI) and 180-bp centromeric repeats. Both are highly methylated and either not expressed or practically not expressed in wild-type plants, but become hypomethylated and transcribed in *met*1 or *ddm*1 mutants (Mittelsten Scheid *et al.*, 1998; Steimer *et al.*, 2000; Vongs *et al.*, 1993). To distinguish DNA methylation at CG sites and CHG sites, we used the restriction enzyme *Hpa*II (sensitive to methylation at both cytosine residues in the recognition site CCGG) and its isoschizomere *Msp*I (limited only by ^m^CCGG; McClelland *et al.*, 1994).

**Figure 4:**
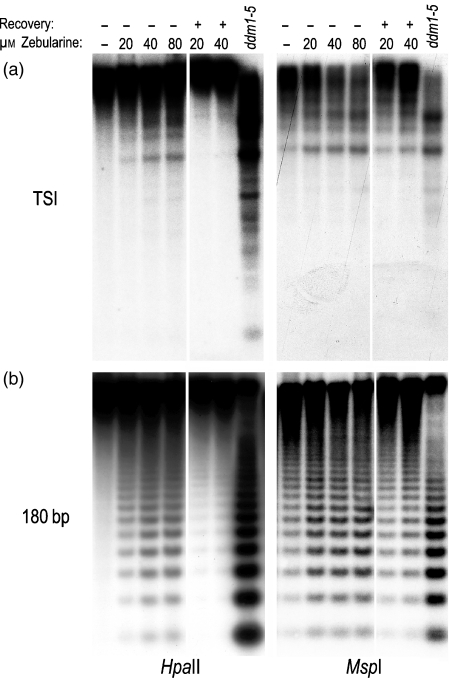
DNA methylation at repetitive sequences is decreased after zebularine treatment Genomic DNA from mock- or zebularine-treated plants and *ddm*1-5 mutants was digested with *Hpa*II and *Msp*I (sensitive to CG and CHG methylation, respectively) and hybridized to (a) transcriptionally silent information (TSI) and (b) 180-bp centromeric (pAL) repeats. Adult plants, recovered for 8 weeks after zebularine treatment, were also included.

As expected, repeat sequences from control plants were not cut by *Hpa*II and only weakly by *Msp*I, indicating strong methylation in both sequence contexts prior to drug treatment. Zebularine-treated plants showed DNA hypomethylation most prominently at CG sites of both TSI and 180-bp repeats, in a concentration-dependent manner (Figure 4a,b). The CHG sites were also affected, but to a lesser extent. Although the total content of 5-mdC in drug-treated plants was reduced to the same low level as in *ddm*1-5 plants, the hypomethylation of TSI and 180-bp repeats at CG and CHG sites was less pronounced than in the mutants. This indicates that the effects of zebularine are not biased towards demethylation of repetitive sequences, in contrast to the effect of the *ddm1* mutation (Vongs *et al.*, 1993).

While the restoration of DNA methylation patterns at repetitive regions can take several generations after outcrossing the *ddm*1 mutation (Kakutani *et al.*, 1996), methylation at TSI repeats is essentially restored in plants that were allowed to recover for 8 weeks after zebularine treatment (Figure 4a). The same was observed at 180-bp repeats, although prolonged exposure of the blots showed some minor remnants of demethylated repeats in recovered plants (Figure 4b).

### Zebularine causes dispersion of heterochromatic chromocentres but not complete depletion of 5-mdC

**Figure 5:**
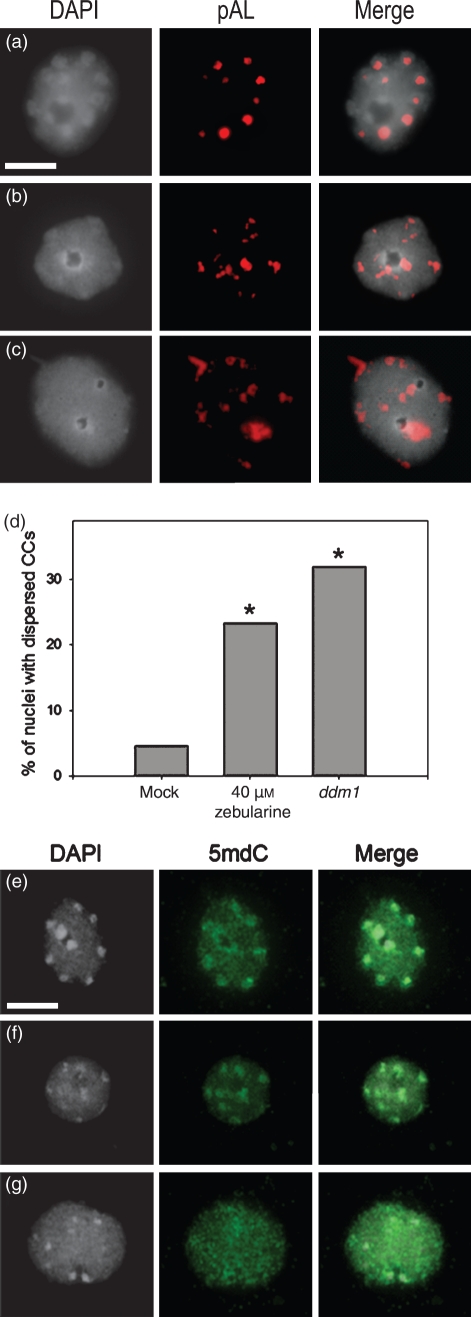
Morphology of centromeric repeats and distribution of 5-methyldeoxycytosine (5-mdC) in zebularine-treated nuclei Cytological analysis by fluorescent *in situ* hybridization (FISH) with centromeric repeats (180 bp, pAL) revealed nuclei with either compact or dispersed signals, the first type representative of nuclei from mock-treated plants (a), the latter characteristic of nuclei from plants treated with 40 μm zebularine (b) or *ddm*1 mutants (c). (d) Nuclei with dispersed chromocentres are five-fold and seven-fold more abundant after treatment with 40 μm zebularine (*n* = 150) and in *ddm*1 mutant plant (*n* = 50) nuclei when compared with mock-treated nuclei (*n* = 150) (*t*-test, *P < 0.001). Immunolocalization of 5-mdC shows an unchanged distribution and signal intensity in (e) mock-treated and (f) 40 μm zebularine-treated nuclei, regardless of their dispersed chromocentres. (g) *ddm*1 nuclei display a strong reduction of DNA methylation at the chromocentres; however, gene body methylation is visible as uniform staining of euchromatin and seems not to be affected. Bars, 5 μm. DAPI, 4′,6-diamidino-2-phenylindole.

Centromeric and pericentromeric repeats in Arabidopsis form heterochromatin that remains strongly condensed in interphase nuclei. These chromocentres (CCs) become decondensed and diffuse upon hypomethylation at centromeric repeats in *ddm*1 mutants (Probst *et al.*, 2003; Soppe *et al.*, 2002). Fluorescence *in situ* hybridization on nuclei from plants treated with 40 μm zebularine indeed contained less prominent and more dispersed CCs, as in *ddm*1 (Figure 5a–c), and these were significantly more frequent in zebularine-treated samples (25%) versus mock treatment (5%), and in a similar range as in *ddm*1 (34%) (Figure 5d). Thus, zebularine treatment causes similar changes in CC morphology as the *ddm*1 mutation.

While 5-mdC seems to be nearly erased from the residual condensed chromatin in *ddm*1, as seen upon immunostaining, the modification is still prominent at the remaining CCs in the drug-treated samples (Figure 5e–g). This is in accordance with the different degree of demethylation at the centromeric repeats seen at the molecular level for *ddm*1 and zebularine treatment (Figure 4). However, the limited loss of methylation by zebularine apparently seems sufficient to loosen condensation of the CCs, and the presence of 5-mC immunofluorescence signals in CCs adds to the evidence that zebularine induces a rather unbiased loss of DNA methylation throughout the genome.

### Zebularine causes reactivation of transcriptionally inactive endogenous loci

**Figure 6:**
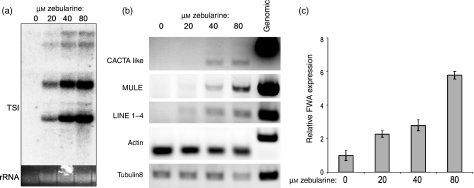
Zebularine-dependent reactivation of transcriptionally silenced genes (a) Northern blot analysis for transcriptionally silent information (TSI) mRNA accumulation after zebularine treatment. (b) The RT-PCR assay for CACTA-like, MULE and LINE1-4 transposon reactivation after zebularine treatment. Actin and tubulin transcripts were used as loading controls. (c) Abundance of *FWA* transcript in relation to Elongation Initiation Factor 4A (*EIF*4A) mRNA in pooled seedlings measured in triplicate by RT-qPCR.

Perturbation of DNA methylation by genetic means or by inhibitors is frequently associated with transcriptional reactivation of otherwise hypermethylated sequences, such as repetitive endogenous sequences or some transgenes. Plant transposons are tightly regulated by the DNA methylation machinery to prevent replication and further spreading throughout the plant genome (Zilberman and Henikoff, 2004). Their transcription can serve as indicators for interference with methylation (Jeddeloh *et al.*, 1998, 1999; Kankel *et al.*, 2003). Therefore, we analysed plants grown on increasing dosages of zebularine for transcriptional activity of TSI and different transposons. Increasing amounts of zebularine led to a dose-dependent release of silencing at TSI loci and up-regulation of CACTA-like and MULE transposons as well as the LINE1-4 non-long terminal repeat (LTR) retrotransposon (Figure 6a,b). The expression of *ACTIN* and *TUBULIN8* was not affected by zebularine treatment (Figure 6b), allowing these genes to serve as loading controls.

Endogenous single-copy genes have also been reported to be regulated by DNA methylation, such as the imprinted *FWA* gene that is methylated in the promoter region and not expressed in vegetative plant tissues (Soppe *et al.*, 2000). However, *FWA* expression is induced in *ddm*1 and *met*1 mutants (Kakutani, 1997; Soppe *et al.*, 2000). We analysed *FWA* expression in zebularine-treated plants by quantitative RT-PCR and observed a dose-dependent increase in *FWA* mRNA levels after zebularine treatment. The highest dose resulted in a six-fold up-regulation compared with mock-treated plants (Figure 6c). Thus, zebularine treatment can induce transcriptional activity at repetitive and single-copy sequences that are otherwise hypermethylated and not expressed.

### Zebularine treatment affects DNA methylation of CG, CHG and CHH sites

**Figure 7:**
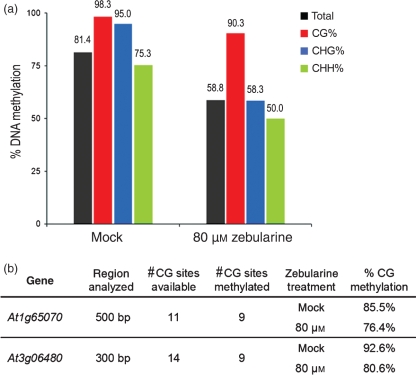
Zebularine treatment reduces *FWA* promoter methylation and genic methylation (a) Total and sequence context-specific DNA methylation determined by bisulphite sequencing of eight clones representing the *FWA* promoter and (b) CG methylation at two coding regions of genes with gene body methylation after treatment with 80 μm zebularine (8 and 12 clones, respectively).

The data described above indicated that the demethylating and transcriptionally reactivating effect of zebularine did not discriminate between the location of 5-mdC within repetitive sequences or single-copy genes. To further investigate whether the effect was also independent of the directly adjacent sequence context and whether zebularine inhibits all methyltransferases equally, we investigated the loss of DNA methylation after drug treatment by bisulphite conversion and sequencing. To focus the analysis on a sequence with a well-defined methylation pattern, we chose one of the short interspersed nucleotide element (SINE)-related direct repeats at the *FWA* gene, which is silent during the vegetative phase of Arabidopsis (Kinoshita *et al.*, 2007; Soppe *et al.*, 2000). Bisulphite sequencing can detect DNA methylation at every cytosine residue in a given sequence with high resolution. Bisulphite conversion was performed on DNA obtained from seedlings that were grown for 3 weeks on 80 μm zebularine, with mock-treated plants of the same age as controls. Total DNA methylation was reduced in zebularine-treated plants to 58.8% of all available sites, compared to 81.4% in untreated wild-type plants. The CHG and CHH methylation data published previously for the same sequence (http://epigenomics.mcdb.ucla.edu/DNAmeth/) (Cokus *et al.*, 2008) are slightly lower, probably reflecting an ecotype-dependent methylation polymorphism. However, zebularine treatment affected all sites: for CG from 98.3–90.3%, for CHG from 95–58.3% and for CHH from 75.3–50% (Figure 7a). With methylation in mock-treated plants set at 100%, the drug application reduced relative values by 8.1% (CG), 38.7% (CHG) and 33.6% (CHH). Thus, demethylation by zebularine appears to be unbiased with regard to the sequence context and seems to affect all methyltransferases.

Since zebularine was more effective than the *ddm*1 mutation with regard to global methylation, but induced less demethylation at repetitive sequences than the mutation, we asked whether the substantial methylation at coding regions of many genes would be affected. We extended the bisulphite sequence analysis to two genes that contain CG-specific gene body DNA methylation (Zilberman *et al.*, 2007) which is reduced in a *met*1 mutant background (Zhang *et al.*, 2006). A MutS DNA mismatch repair gene (At1g65070) and a RNA helicase (At3g06480) have 85.5% and 92.6% CG site-specific methylation, respectively, in mock-treated plants. After 80 μm zebularine treatment, these values are reduced by 23.6% and 19.4% CG methylation for At1g65070 and At3g06480, respectively (Figure 7b). Zebularine therefore induces hypomethylation at all types of sequences, in an unbiased manner and apparently in proportion to the degree of pre-existing methylation.

### Zebularine induces reactivation of transcriptionally inactive transgenic loci

**Figure 8:**
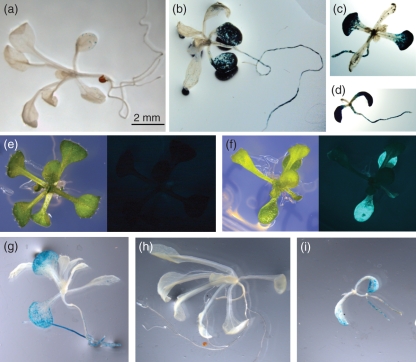
Effects of zebularine and/or trichostatin A on repetitive transcriptionally silent transgenes Transgenic lines with repetitive, transcriptionally silent GUS (TS-GUS) (a–d) genes are reactivated by (b) 20 μm, (c) 40 μm and (d) 80 μm zebularine. Additionally, a line containing transcriptionally silent GFP (TS-GFP; e, f) genes is reactivated by (f) 40 μm zebularine. Mock-treated plants (a) and (e) were used as controls. TS-GUS seedlings, 3 weeks old after treatment with (g) 40 μm zebularine, (h) 40 μm trichostatin A and (i) a combination of both.

Changes in epigenetic regulation are frequently analysed based on reporter genes whose expression can be visualized by enzymatic staining reactions or fluorescence. In plants, the β-glucuronidase reporter (GUS) and green fluorescent protein (GFP) are widely used reporter, and transgenic lines with transcriptionally silenced marker genes are available for both. TS-GUS (6b5/L2, (Morel *et al.*, 2000; Probst *et al.*, 2004)) and TS-GFP (L5, T. Blevins and F. Meins, Friedrich Miescher Institute for Biomedical Research, Basel, Switzerland, pers. comm.) contain repetitive GUS or GFP genes, respectively, which had been shown previously to become reactivated in the background of mutants affecting DNA methylation and chromatin remodelling, such as *ddm*1-5, *met*1-3 or *mom*1-1 (Amedeo *et al.*, 2000; Morel *et al.*, 2000; T. Blevins, pers. comm.). To visualize reactivation by zebularine-induced DNA demethylation *in planta*, seedlings of lines TS-GUS and TS-GFP were grown for 21 days on plates containing zebularine prior to analysis for GUS and GFP expression. Mock-treated seedlings showed neither significant GUS staining nor GFP expression (Figure 8a,e), whereas the zebularine treatment released silencing of TS-GUS at concentrations of 20, 40 and 80 μm (Figure 8b–d). The TS-GFP plants, pre-treated with 40 μm zebularine, were also positive for transgene expression (Figure 8f).

The methylation inhibitor 5-aza had been shown to act synergistically in combination with trichostatin A (TSA), a histone de-acetylase inhibitor affecting gene silencing in animals and plants (Chen and Pikaard, 1997; Gartler and Goldman, 1994), although the interaction in plants is complex and can be antagonistic for certain target genes (Chang and Pikaard, 2005). We therefore tested zebularine in combination with TSA. The TS-GUS and TS-GFP seeds were germinated on media with either 1.6 μm (0.5 μg ml^−1^) TSA or 40 μm zebularine or both drugs at the same concentration as for the single treatments. Trichostatin A alone did not reactivate the silent reporter *GUS* gene even after 3 weeks of application (Figure 8h). A synergistic effect of TSA and zebularine was observed on plant growth and development, which were inhibited since seedlings treated with both drugs were much smaller than mock-, TSA- or zebularine-treated seedlings. However, the effect of the drug combination upon reporter gene expression seemed to be rather the opposite, because staining in TS-GUS plantlets was less intense than with zebularine treatment alone (Figure 8i). This might be due to the general growth inhibition that could reduce the potential for GUS and GFP expression, or indicate antagonistic effects between histone deacetylase inhibitors and 5-mdC inhibitors similar to those reported earlier (Chang and Pikaard, 2005).

## Discussion

Methylation of cytosine residues is the most frequent chemical modification of genomic plant DNA and is found in such amounts that the terminology of the ‘fifth nucleotide’ (Doerfler, 2006) is as justified in this kingdom as for mammalian DNA. 5-Methyldeoxycytidine is an important element of epigenetic regulation in plants, diverse with regard to sequence context, location at gene bodies or non-coding regions, single-copy sequences or repeats. It is a stable, covalent modification, yet amenable to addition or removal by enzymatic activities or to passive loss upon replication or loss of functional methyltransferases. Specific methylation inhibitors are considered to be important tools for studying the biological role of DNA methylation, as apparent from the frequent use of the methylation inhibitor 5-aza (Lyko and Brown, 2005). However, this compound has an extremely short half-life in aqueous solutions such as plant growth media, not matching the long culture periods necessary for plant development. Therefore, to achieve reliable and reproducible general demethylation, the potential of zebularine, an agent with a similar mode of action to 5-aza but significantly better chemical stability (Cheng *et al.*, 2003; Zhou *et al.*, 2002), was explored in plant culture, and indeed, the presence of zebularine in the growth medium induced a significant, global reduction of 5-mdC in two plant species. In a direct comparison, zebularine caused an even slightly higher global demethylation than 5-aza, which can be due to better uptake, better integration or most likely due to higher stability in the plant culture medium. We showed that, in Arabidopsis, zebularine induced a non-discriminative and dosage-dependent reduction of 5-mdC. This offers certain advantages over the use of genetic mutants affecting DNA methylation only in certain sequence contexts such as ^m^CG, ^m^CHG or ^m^CHH, or restricted to certain chromosomal regions and targets.

The preferential loss of methylation at centromeric regions in *ddm*1 mutant nuclei causes a significant decondensation and dispersion of the centromeric heterochromatin. The hypomethylation by zebularine is much less pronounced at centromeric repeats, as is apparent from molecular and cytological analysis. Nevertheless, *ddm*1 mutant nuclei and inhibitor-treated material showed a similar change in nuclear organization. This indicates that small changes in the methylation level are sufficient to interfere with the maintenance of the condensed state. Alternatively, the methylation status of other regions may contribute to condensation of heterochromatic regions, by recruiting interacting proteins or shaping larger complexes of nuclear organization. A direct or indirect effect of demethylation on nuclear organization at the chromosome level has also been observed for centromeres in polyploid wheat: the somatic association of homologous as well as homeologous centromeres was significantly reduced in xylem vessel cells upon treatment of roots with 5-aza (Vorontsova *et al.*, 2004).

Loss of DNA methylation upon genetic interference can become more drastic over several generations of inbreeding homozygous mutants (Kakutani *et al.*, 1996) or persist into subsequent generations even upon restitution of the methylation machinery after outcrossing with wild-type plants (Kakutani *et al.*, 1999). Data for application of 5-aza are not unambiguous. While there is a claim for heritable demethylation and morphological consequences in progeny of treated rice seedlings (Sano *et al.*, 1990), other studies have shown a transient effect (Kumpatla and Hall, 1998). Conversely, the demethylating effect of zebularine is transient, since DNA methylation level and patterns are restored in somatic tissue formed after removal of the drug. This suggests that the blueprint for the methylation patterns is not fully removed. It could either reside in the residual methylation itself or in some other chromatin-associated information that may be erased by the mutations but not by zebularine. Extension of the methylation analysis to both strands of the same genomic template by hairpin bisulphite sequencing (Laird *et al.*, 2004) could permit investigation into how far the methylation is erased from both Cs at symmetric methylation sites. Together with pulsed application of zebularine-induced demethylation, this will allow an analysis of the pre-requisites and kinetics of remethylation.

The response of transcriptionally silenced targets to zebularine treatment was crucial to claim an equal or superior action of this drug. This has been proven for several endogenous indicators (centromeric repeats and transposons) and repetitive transgenic marker genes (TS-GUS, TS-GFP) as well as for protein-coding genes that are under transcriptional control of neighbouring low-copy repeats (*FWA*). Their dose-dependent reactivation after zebularine treatment seems to be directly connected with the dose-dependent demethylation. Interestingly, the three transposons included in our study respond in a similar way (although to different levels; Figure 6). This is not the case upon genetic interference with methylation: while retrotransposon LINE1-4 is significantly activated in a *ddm*1, *met*1 and *cmt*3 mutant background, the Mule transposon is not up-regulated in *cmt*3 (Lippman *et al.*, 2003). This is further evidence that zebularine discriminates less between different methylation types and targets. Data about release of these transposons from silencing by treatment with 5-aza are not available, since they were underrepresented on the microarrays used in the otherwise most comprehensive study of Chang and Pikaard (2005). However, a direct comparison of the two drugs in human cell culture indicated that both could reactivate a methylated gene relevant for cell adhesion and invasiveness, while 5-aza treatment (even at a much lower dose) additionally activated a latent virus (Rao *et al.*, 2007). This may indicate a different spectrum of action and allows a fine-tuned application of zebularine for specific experimental purposes.

## Experimental procedures

### Plant growth and chemical treatments

Cold-treated seeds were sterilized in 5% sodium hypochlorite and 0.05% Tween-80 for 6 min, washed and air-dried overnight. Sterilized seeds were sown and grown directly onto Petri dishes with agar-solidified germination medium containing zebularine (Sigma, http://www.sigmaaldrich.com/), 5-aza (Sigma) and/or TSA (Sigma) and grown for 21 days in growth chambers under 16-h light/8-h dark cycles at 21°C. Zebularine and 5-aza in aqueous solution or TSA dissolved in DMSO were added to the germination medium before solidifying at final concentrations of 20, 40 and 80 μm of zebularine, 40 μm 5-aza and 1.6 μm (0.5 μg ml^−1^) of TSA. Plants were transferred to drug-free growth medium after 14 or 21 days for recovery.

### Nucleic acid isolation and gel-blot analysis

Seedlings were harvested as pools of 100 plantlets, shock-frozen in liquid nitrogen and homogenized by vortexing for 1 min using two or three ceramic spheres of diameter 1 cm. Rosette and stem leafs from three to five adult plants were harvested, shock-frozen in liquid nitrogen and homogenized. Homogenized plant tissue was subsequently used for DNA or RNA extraction using Phytopure (Amersham, http://www5.amershambiosciences.com/) or RNAeasy (Qiagen, http://www.qiagen.com/) kits, respectively.

For Southern blot analysis, 10 μg of genomic DNA was digested overnight with 1–2 U *Hpa*II or *Msp*I (MBI Fermentas, http://www.fermentas.com/). Subsequently, samples were electrophoretically separated on 1.2% TRIS–acetate–ethylenediamine tetraacetic acid [TAE; TRIS = 2-amino-2-(hydroxymethyl)-1,3-propanediol] agarose gels, depurinated for 10 min in 250 mm HCl, denatured for 30 min in denaturation solution containing 0.5 m NaOH and 1.5 m NaCl and neutralized twice in 0.5 m TRIS, 1.5 m NaCl and 1 mm EDTA at pH 7.2 for 15 min. For northern blot analysis, 10 μg of total RNA was denatured with 15% glyoxal and DMSO for 1 h at 50°C and separated using 1.4% agarose gels in 10 mm sodium phosphate buffer pH 7 in a Sea2000 circular flow electrophoresis chamber (Elchrom Scientific, http://www.elchrom.com/). DNA and RNA gels were blotted onto Hybond N+ (Amersham) membranes overnight with 20× SSC, washed and UV-crosslinked using a Stratalinker (Stratagene, http://www.stratagene.com/). Hybridization was performed as described by Church and Gilbert (1984). Radioactive (50 μCi) dCT-α-^32^P (Amersham) labelled sequence-specific probes (TSI-A15 and pAL-180 bp) were synthesized from 25 ng of DNA using the Rediprime labelling kit (Amersham) and purified on G50 Probequant (Amersham) columns. Signals were detected with Phosphorimager Screens (Bio-Rad, http://www.bio-rad.com/) and scanned with a Molecular Imager FX (Bio-Rad).

### Cation-exchange high-pressure liquid chromatography

Total cytosine methylation was determined as described (Rozhon *et al.*, 2008). In short, 5 μg of genomic DNA was digested overnight at 37°C with 0.0025 U nuclease P1 and 0.5 U DNaseI in 20 mm acetic acid, 20 mm glycine, 5 mm MgCl_2_, 0.5 mm ZnCl_2_ and 0.2 mm CaCl_2_, pH 5.3 in a total volume of 50 μl. Subsequently, 5 μl of 0.1 m NaOH and 1 U calf intestine alkaline phosphatase were added and the mixture incubated for a further 24 h. Samples were acidified by addition of 44 μl of 12 mm HCl prior to injection into the HPLC system equipped with a 125 × 4 mm Nucleosil 100-10 SA column (Macherey-Nagel, http://www.macherey-nagel.com/) preceded by a Valco 2 μm inline filter. The mobile phase consisted of 60 mm acetic acid and 15% acetonitrile, pH 4.8, with a constant flow rate of 1.5 ml min^−1^. Ultraviolet detection was performed at 277 nm with a bandwidth of 10 nm with a PDA-100 photodiode array detector, and chromatograms were analyzed with Chromeleon 7 (Dionex, http://www.dionex.com/). All samples were analysed in technical triplicates and 5-mdC values were expressed as a percentage of total cytosine.

### Reverse transcription PCR and real-time PCR

Prior to reverse transcription, 30 μl RNA solution was treated with 5 U DNase I (MBI Fermentas), 0.4 U ribonuclease inhibitor (Rnasin) and 4 μl of 10× DNase I buffer for 40 min at 37°C to remove residual DNA contamination in the RNA samples, extracted with phenol:chloroform (24:1) and subsequently ethanol-precipitated. Reverse transcription was performed on 1 μg of RNA with 0.2 μg random hexamer primers (MBI Fermentas) using 1 U RevertAid M-MuLV-RTase, RNaseH- (MBI Fermentas) at 42°C for 1.5 h. The cDNA thereby obtained was used for PCR and real-time PCR. Standard PCR was performed with True-Start Taq polymerase (Promega, http://www.promega.com/) and the following primers: CACTA-F: 5′-GGCTAGCTGTCCGACTCAATGACCT-3′, CACTA-R: 5′-CAGACATCCTTTCCTTCAGCTTAGC-3′, MULE2-F: 5′-CTGTCCGCGAGTGTCATCAAGTAGC-3′, MULE2-R: 5′-GATACTTGTTGACAAGTGTTTAGCAAGCC-3′, FWA-RTF: 5′-GTGTTAATGATCAAGATGGTGGAA-3′, FWA-RTR: 5′-AAGCTCGTACCTCTGTTCTTCAGT-3′, ActinF: 5′-TCCCTCAGCACATTCCAGCAGAT-3′, ActinR: 5′-AACGATTCCTGGACCTGCCTCATC-3′, SN1F: 5′-ACTTAATTAGCACTCAAATTAAACAAAATAGT-3′, SN1R: 5′-TTTAAACATAAGAAGAAGTTCCTTTTTCATCTAC-3′, EIF4A-F: 5′-ATCCAAGTTGGTGTGTTCTCC-3′ and EIF4A-R: 5′-GAGTGTCTCGAGCTTCCACTC-3′. Real-time PCR analysis was performed with the DyNAmo SYBRgreen kit (New England Biolabs, http://www.neb.com/) using a Rotorgene 3000 (Corbett, http://www.corbettlifescience.com/) lightcycler with data acquisition at 72°C to avoid signals from primer dimers. Ct values were analysed using Excel (Microsoft, http://www.microsoft.com/).

### *In situ* GUS and GFP detection

The GUS activity was detected by staining in 0.1 m sodium phosphate buffer pH 7.0, 10 mm EDTA, 0.1% Triton X-100, 100 μg ml^−1^ chloramphenicol, 2 mm potassium ferrocyanide, 2 mm potassium ferricyanide and 0.5 mg ml^−1^ X-Gluc after 30-min vacuum infiltration and overnight incubation at 37°C. Subsequent washes with 70% ethanol at 37°C were performed in order to remove residual chlorophyll. All samples were analysed using a Leica MZ16FA binocular microscope with a Leica DFC300FX CCD camera (http://www.leica.com/). Images were acquired with Leica Application Suite and processed with Adobe Photoshop (Adobe, http://www.adobe.com/). Plants transgenic for TS-GFP were analysed under UV illumination with a Leica GFP1 filter (excitation 425/60 nm, emission barrier 480 nm) directly on plates.

### Fluorescence *in situ* hybridization (FISH) and immunolabelling detection

For the preparation of nuclei, 21-day-old plantlets were rinsed in 10 mm TRIS buffer pH 7.5, fixed by vacuum infiltration in 4% formaldehyde/TRIS buffer, rinsed in TRIS buffer, chopped in 500 μl chromosome isolation (CI) buffer (15 mm TRIS, 2 mm Na2EDTA, 0.5 mm spermin, 80 mm KCl, 20 mm NaCl, 15 mm beta-mercaptoethanol, 0.1% Triton X-100, pH 7.5) and filtered through a 50-μm nylon mesh. Fifty microlitres of nuclei suspension was transferred onto microscope slides using Cytospin (560 **g** for 10 min). After centrifugation, slides were shortly rinsed in 1× PBS, transferred into 50% glycerol and stored at −20°C until use.

Immunolocalization of methylated cytosine was performed as described (Jasencakova *et al.*, 2000) with minor modifications. In brief, slides were treated with pepsin (50 μg ml^−1^ in 0.01 m HCl; Roche, http://www.roche.com/) at 38°C (1–2 min), post-fixed in 4% formaldehyde/2× SSC, denatured in 70% formamide/2× SSC at 80°C (2 min) and cooled in ice-cold 1× PBS. After blocking (5% BSA, 0.2% Tween 20, 4× SSC) at 37°C (30 min), the slides were incubated with primary monoclonal mouse-anti-5-methylcytosine (1:500, Eurogentec, http://www.eurogentec.com/) and secondary goat-anti-mouse-Alexa488 (1:250, Molecular Probes, http://www.invitrogen.com/site/us/en/home/brands/Molecular-Probes.html) antibodies.

A biotin-labelled Arabidopsis centromeric repeat (pAL, 180 bp) probe for FISH was prepared from genomic DNA by PCR using primers pALU 5′-AGTCTTTGGCTTTGTGTCTT-3′ and pALR 5′-TGGACTTTGGCTACACCATG-3′. Slide pre-treatment and detection steps were performed as described (Pecinka *et al.*, 2004). The probe was detected with subsequent avidin-Texas Red (1:1000, Vector Laboratories, http://www.vectorlabs.com/), goat-anti-avidin-biotin (1:200, Vector Laboratories) and again avidin-Texas Red (1:1000). The slides were counterstained with 4′,6-diamidino-2-phenylindole (DAPI) [1 μg ml^−1^ in Vectashield (Vector Laboratories)] and analysed using a Zeiss Axioplan 2 epifluorescence microscope. Monochromatic images were acquired with MetaVue (http://www.moleculardevices.com/pages/software/metavue.html) and processed with Adobe Photoshop (Adobe).

### Bisulphite conversion, sequencing and evaluation

After treatment with RNaseA and proteinase K, 1–2 μg of genomic DNA was digested overnight with *Bam*HI (MBI Fermentas). Subsequent bisulphite conversion was carried out using the Epitect Conversion Kit (Qiagen) and controlled for completion as described (Hetzl *et al.*, 2007). Converted DNA was used for PCR amplification with the following primer pairs: FWA-L1: 5′-GGGTTTAGTGTTTAYTTGTTTAAGG-3′, FWA-R4: 5′-TCTRATTRTCARTATCCCACAAATC-3′, At1g65070bsF: 5′-GTATYYGTGAGATGTGGTTATTAAAGGTTG-3′, At1g65070bsR: 5′-CATCACATACAAATTAAATTAATAATATCTATCCC-3′, At3g06480bsF: 5′-GAAGTAGTATAAATAYGAATAAAGGTAAGTAATTTTG-3′ and At3g06480bsR: 5′-CTRAAACAAACCCATCCTTATAACRCARTATATT-3′ (Zilberman *et al.*, 2007). The PCR-amplified DNA was cloned using CloneJet or InsTAclone kits (MBI Fermentas) and transformed into DH5α cells (Invitrogen), sequenced by terminal labelling using BigDye Terminator v3.1 (Applied Biosystems, http://www.appliedbiosystems.com/) and read at http://www.vbc-genomics.com. The sequence information obtained was analysed with CyMATE (http://www.gmi.oeaw.ac.at/cymate; Hetzl *et al.*, 2007) and Excel (Microsoft).
